# Supplementary material for: Identification and functional analysis of non-coding regulatory small RNA FenSr3 in Bacillus amyloliquefaciens LPB-18
Source: PeerJ. 2023 May 15;11:e15236. doi: 10.7717/peerj.15236 (PMC10194069; doi:10.7717/peerj.15236)
Supplement: Supplemental Information 4 [file peerj-11-15236-s004.zip › KO/CK-vs-T1_map/map01110.html]

KEGG PATHWAY: Biosynthesis of secondary metabolites - Reference pathway


|  |  |
| --- | --- |
| **Biosynthesis of secondary metabolites - Reference pathway** |  |

[
Pathway menu
| Organism menu
| Pathway entry
|

Hide module list

| User data mapping
|

Image (png) file

]

|  |  |  |
| --- | --- | --- |
| Reference pathway | 100% 70% 35% | 图片下载 |

- **Category title**

  

- **KEGG module**

- Carbohydrate and lipid metabolism
  - Other carbohydrate metabolism
    - M00012 Glyoxylate cycle- M00532 Photorespiration
      - M00565 Trehalose biosynthesis, D-glucose 1P => trehalose
  - Fatty acid metabolism
    - M00113 Jasmonic acid biosynthesis
  - Terpenoid backbone biosynthesis
    - M00095 C5 isoprenoid biosynthesis, mevalonate pathway- M00849 C5 isoprenoid biosynthesis, mevalonate pathway, archaea
      - M00096 C5 isoprenoid biosynthesis, non-mevalonate pathway
      - M00364 C10-C20 isoprenoid biosynthesis, bacteria
      - M00365 C10-C20 isoprenoid biosynthesis, archaea
      - M00366 C10-C20 isoprenoid biosynthesis, plants
      - M00367 C10-C20 isoprenoid biosynthesis, non-plant eukaryotes
  - Other terpenoid biosynthesis
    - M00097 beta-Carotene biosynthesis, GGAP => beta-carotene- M00372 Abscisic acid biosynthesis, beta-carotene => abscisic acid
      - M00371 Castasterone biosynthesis, campesterol => castasterone
- Nucleotide and amino acid metabolism
  - Serine and threonine metabolism
    - M00018 Threonine biosynthesis, aspartate => homoserine => threonine
  - Cysteine and methionine metabolism
    - M00021 Cysteine biosynthesis, serine => cysteine- M00368 Ethylene biosynthesis, methionine => ethylene
  - Branched-chain amino acid metabolism
    - M00019 Valine/isoleucine biosynthesis, pyruvate => valine / 2-oxobutanoate => isoleucine- M00432 Leucine biosynthesis, 2-oxoisovalerate => 2-oxoisocaproate
  - Lysine metabolism
    - M00527 Lysine biosynthesis, DAP aminotransferase pathway, aspartate => lysine
  - Histidine metabolism
    - M00026 Histidine biosynthesis, PRPP => histidine
  - Aromatic amino acid metabolism
    - M00022 Shikimate pathway, phosphoenolpyruvate + erythrose-4P => chorismate- M00023 Tryptophan biosynthesis, chorismate => tryptophan
      - M00024 Phenylalanine biosynthesis, chorismate => phenylalanine
      - M00040 Tyrosine biosynthesis, prephanate => pretyrosine => tyrosine
      - M00350 Capsaicin biosynthesis, L-Phenylalanine => Capsaicin
  - Cofactor and vitamin biosynthesis
    - M00125 Riboflavin biosynthesis, GTP => riboflavin/FMN/FAD- M00119 Pantothenate biosynthesis, valine/L-aspartate => pantothenate
      - M00121 Heme biosynthesis, glutamate => heme
      - M00846 Siroheme biosynthesis, glutamate => siroheme
      - M00847 Heme biosynthesis, archaea, siroheme => heme
      - M00114 Ascorbate biosynthesis, plants, glucose-6P => ascorbate
      - M00112 Tocopherol/tocotorienol biosynthesis
      - M00117 Ubiquinone biosynthesis, prokaryotes, chorismate => ubiquinone
      - M00116 Menaquinone biosynthesis, chorismate => menaquinone
- Secondary metabolism
  - Biosynthesis of secondary metabolites
    - M00039 Monolignol biosynthesis, phenylalanine / tyrosine => monolignol- M00137 Flavanone biosynthesis, phenylalanine => naringenin
      - M00138 Flavonoid biosynthesis, naringenin => pelargonidin
      - M00369 Cyanogenic glycoside biosynthesis, tyrosine => dhurrin
      - M00370 Glucosinolate biosynthesis, tryptophan => glucobrassicin
      - M00661 Paspaline biosynthesis, geranylgeranyl-PP + indoleglycerol phosphate => paspaline
